# First steps in the development of an ovine proximal phalanx fracture and bone defect model: a study of animal welfare and bone healing

**DOI:** 10.3389/fvets.2025.1662553

**Published:** 2025-10-08

**Authors:** Nanett Kvist Nikolaisen, Thomas Colding-Rasmussen, Peter F. Horstmann, Anna V. Müller, José Joaquín Cerón, Michael Mørk Petersen, Christian Nai En Tierp-Wong, Stine Jacobsen

**Affiliations:** ^1^Department of Veterinary Clinical Sciences, Faculty of Health and Medical Sciences, University of Copenhagen, Copenhagen, Denmark; ^2^Department of Orthopaedic Surgery, Hvidovre University Hospital, Copenhagen, Denmark; ^3^Department of Orthopaedic Surgery, Rigshospitalet University Hospital, Copenhagen, Denmark; ^4^Department of Orthopaedic Surgery, Herlev/Gentofte University Hospital, Copenhagen, Denmark; ^5^Department of Animal Medicine and Surgery, Faculty of Veterinary Medicine, University of Murcia, Murcia, Spain

**Keywords:** biomarkers, bone healing, large animal model, pain scores, sheep, small tubular bones

## Abstract

There is a need for large-animal fracture models focusing on small tubular bones, as existing models typically involve major weight-bearing bones and often rely on restrictive suspension systems that raise significant animal welfare concerns. This study presents a novel *in vivo* sheep model targeting the proximal phalanx, designed to enable natural movement and social housing whilst supporting the investigation of fracture fixation and bone healing. Eleven skeletally mature Texel sheep were included; four underwent bilateral ostectomies with 3 mm or 6 mm defects, and seven received an osteotomy. A hoof block was used to offload the affected digit, enabling the sheep to move freely and to be housed in pairs. Bone healing was assessed using standardised radiographic scoring based on images obtained intraoperatively, at week 1 and 2, and hereafter every second week. Hard callus thickness was assessed at the end of the study period by a computed tomography-based method. Animal welfare was monitored through repeated clinical evaluations, two pain scoring systems (a validated sheep facial pain expression scale and a novel composite behavioural pain score), and biomarkers of inflammation, including serum amyloid A and haptoglobin. The ostectomy group showed frequent implant failure and limited healing, particularly in the 6 mm ostectomies, whilst the osteotomy group demonstrated relatively better stability and more consistent healing. Pain scores peaked shortly after surgery and again following withdrawal of analgesia but remained generally low. This study presents a novel, welfare-consciousness bilateral ovine proximal phalanx fracture model that challenges osteosynthesis stability. The integrated multimodal welfare assessment highlights the importance of objective pain and stress monitoring and advocates for the routine inclusion of quantitative welfare parameters alongside bone healing outcomes in translational orthopedic research.

## 1 Introduction

Fractures of non-weight bearing, small tubular bones, such as the phalanges and metacarpals, are common traumatic injuries (1, 2). These injuries often affect younger, working-age individuals and are associated with significant functional impairment and high healthcare costs (3, 4). The treatment can be challenging and associated with a high risk of complications, including stiffness, malunion, and non-union, and reoperations to remove implants are frequent (5, 6). In addition to trauma, other pathologies affecting these bones, such as infections, tumours and segmental bone defects of various causes, also present significant clinical challenges (7–9). Accordingly, there is a need to optimise the treatment of fractures, pathologies and defects in low load bearing small tubular bones.

To study and improve the treatment of bone fractures and bone pathologies in general, large animal models play an essential role. They enable preclinical evaluations of novel materials and techniques as well as advancements in treatment strategies (10, 11). Sheep are widely used due to their similarities to human bone in size, structure, biomechanics, and remodelling behaviour (10). Several fracture and defect models have been established and applied in sheep involving larger load bearing tubular bones such as the femur and tibia (12, 13). The translational relevance of these models to small tubular bones such as the phalanges and metacarpals is limited. This is due to fundamental differences in bone size, biomechanical loading conditions, and healing mechanics, as well as differences in soft tissue coverage and vascularity, with phalanges having minimal muscle coverage. Moreover, fractures in phalanges and metacarpal bones demand rapid rehabilitation to preserve fine motor function and prevent stiffness, which requires a fundamentally different treatment approach compared to long bone injury (14, 15).

Existing ovine fracture models require the protection of the fractured long bone. This is achieved through casts (12, 16–18) and/or by the use of restrictive devices such as slings, where the sheep are suspended for several (up to 9) weeks postoperatively (12, 16, 17, 19–25). This severely limits natural movement and behaviour and raises animal welfare concerns. There is a constant drive towards improving experimental animal welfare (26–28), and the development of fracture models that exert less strain on the experimental animals is highly needed. Measures aimed at minimising model severity and invasiveness, as well as methods improving postoperative welfare monitoring and care, we find are key in making fracture model research more ethically acceptable and valid.

A major societal concern pertains to the use of experimental animals in medical research, including research on fracture healing and repair. In the ARRIVE (Animal Research: Reporting of *In Vivo* Experiments) guidelines 2.0, which is an overview of information to include in publications describing animal research, the recommended set of items to be reported in the scientific paper includes housing and husbandry, care, monitoring, and analgesia (27). Some studies involving sheep in orthopaedic research have assessed pain-related welfare; Häger et al. (21) presented quantitative results from an assessment of pain-related facial expressions and clinical severity scores. Other studies have utilised gait evaluation and/or assessing pain-related behaviour, albeit without reporting quantitative results (16, 25, 29, 30).

To our knowledge, no large animal low load bearing small tubular bone model exists. Accordingly, the aim of this study was to develop an ovine phalanx fracture model. The welfare of the experimental animals was addressed by adopting an armamentarium of methods for monitoring pain and abnormal behaviours and applying them rigorously throughout the study period. Here we report the first steps towards development of a bilateral proximal phalanx fracture model in sheep.

## 2 Methods

### 2.1 Study overview

**Figure 1:**
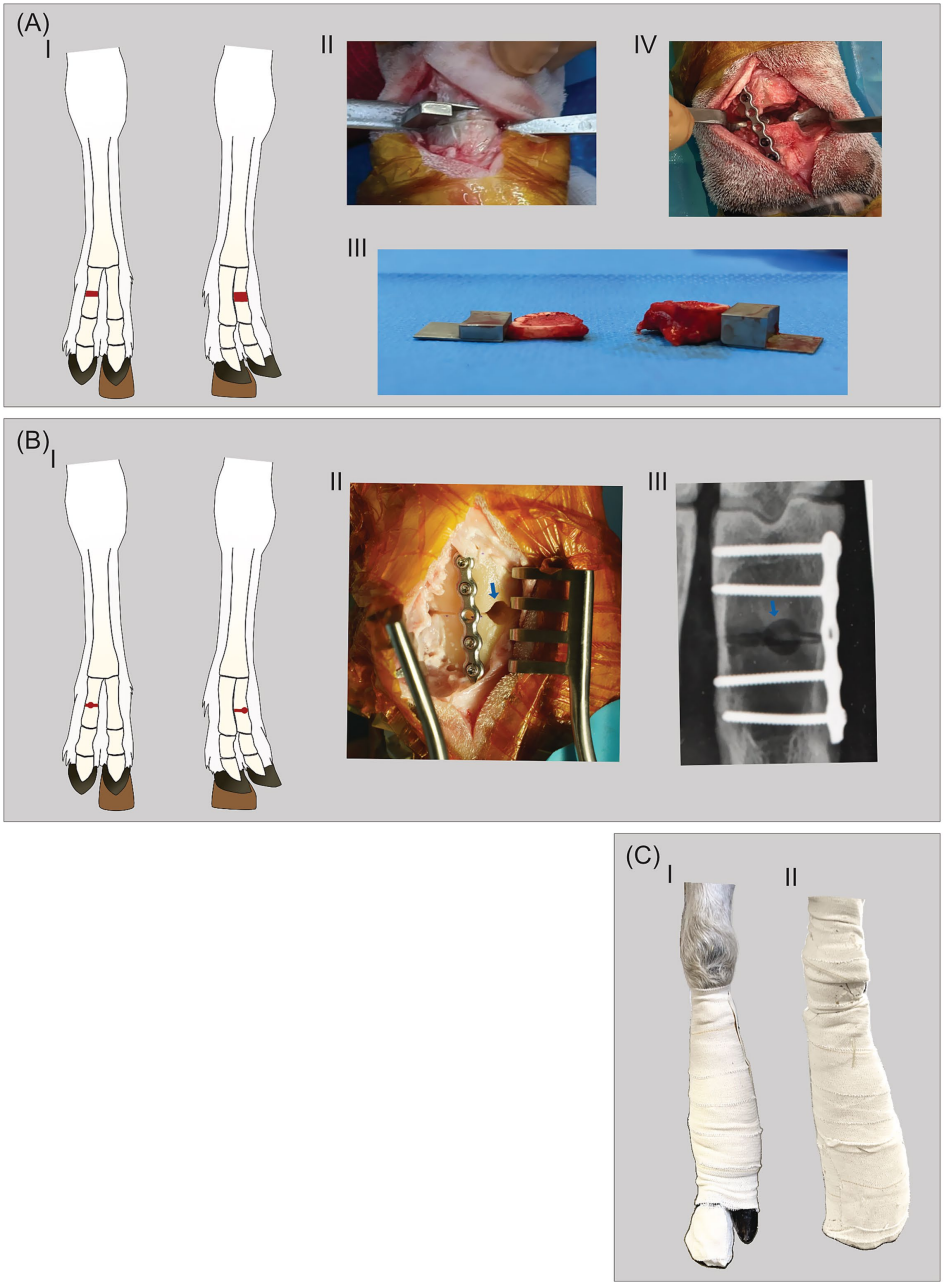
Overview of the ovine thoracic limb bilateral single toe (OBST) fracture model. **(A)** The ostectomy group showing (I) a schematic drawing of the bilateral intervention, (II) the application of the cutting guide, (III) the ostectomized bone segment of 3 mm and 6 mm and (IV) the ostectomized proximal phalanx osteosynthesized with 6-hole locking plate. **(B)** The osteotomy group showing (I) a schematic drawing of the bilateral intervention, (II) the osteotomized proximal phalanx osteosynthesized with 5-hole locking plate, the 4.5 mm drill-hole evident (blue arrow), and (III) radiograph depicturing the osteotomy line, drill-hole (blue arrow), metal-plate and bicortical screws. **(C)** Coaptation modalities of a left thoracic limb; (I) hoof block and elastic bandage, and (II) a half limb walking cast with no hoof block.

This study represents our initial approach to development of a novel ovine bilateral proximal phalanx fracture model. Twelve sheep were included; and the lateral proximal phalanges of both front limbs underwent fracture surgery. In 8 sheep a novel fracture repair modality was tested in one of the two phalanges, these results are reported elsewhere (31), whilst the remaining 16 phalanges were repaired using metallic implants as described below. The bilateral approach enables within-subject comparisons, reducing animal use in line with the 3Rs of animal experimentation (Replacement, Reduction, and Refinement) (32). With one sheep euthanized on day six due to systemic illness, the final sample was 15 phalanges. The fracture models investigated were transverse proximal phalanx midline ost*ectomy* with 3 mm (*n* = 4 phalanges) or 6 mm (*n* = 4 phalanges) gaps, and midline oste*otomy* with central 4.5 mm drill hole (*n* = 7 phalanges) (Figures 1A,B). Fractures were fixated with one dorsolaterally placed stainless-steel locking plate. The sheep had either a walking cast or a custom-made offloading hoof block combined with bandages as coaptation (Figure 1C). By the end of the study period (16 weeks), the sheep were lightly sedated with xylazine (0.08 mg/kg BW, IV, Xysol Vet., ScanVet Animal Health A/S, Denmark) and butorphanol tartrate (0.04 mg/kg BW, IV, Dolorex, MSD Animal Health A/S, Denmark) and euthanised by pentobarbital (140 mg/kg BW, IV, Euthasol vet., Dechra Veterinary Products A/S, Denmark). Bone healing was evaluated by repeated radiographs throughout the 16-week study period and by post-euthanasia computed tomography (CT) scans. Animal welfare was analysed by repeated clinical examinations, lameness, pain face analysis, and by implementing a modified pain behaviour score as detailed below.

### 2.2 Animals

Eleven skeletally mature Texel ewes (5.5 years ± 3 months, 73 kg ± 7.6 kg) were included. The animals were kept in pairs indoors in 6 m^2^ pens with wood shavings as bedding. Their diet was adjusted according to nutritional requirements, and straw and water were provided *ad libitum*. During a 3–10 weeks acclimatization period prior to surgery, clinical health assessments were conducted following the European Animal Welfare Indicators (AWIN) protocol for sheep (33). Additionally, the sheep were trained by positive reinforcement to undergo clinical procedures, including sample collection, clinical examinations, and lameness assessment when led by a leash, so that these procedures could be conducted with minimal stress. In this acclimatization period, routine blood and faecal analyses were performed to ensure enrolment of only healthy animals, and the sheep were treated with Albendazole (4 mg/kg BW, Valbazen Vet., Zoetis Animal Health ApS, Denmark). To facilitate repeated monitoring of core temperature, a temperature-sensitive chip was inserted intramuscularly in *Musculus omotransversarius*, by identifying the fascial border between *Musculus cleidooccipitalis* and the cervical part of *Musculi trapezii* at the dorsal third of the neck (Global-Ident Bio-Thermo, Allflex, France).

### 2.3 Pre-surgical preparation and anaesthetic protocol

The sheep were initially fasted for 36 h preoperatively (*n* = 8) (34) but based on the frequent occurrence of moderate–severe diarrhoea in the immediate postoperative period, fasting duration was reduced to 24 h (*n* = 4) with no adverse effects during surgery and improved gastrointestinal health post-surgically (35). Water was withheld for 4 h before the scheduled surgery time. Premedication for anaesthesia included intravenous (IV) administration of xylazine hydrochloride (0.05 mg/kg, Xysol Vet., ScanVet Animal Health A/S, Denmark) and butorphanol tartrate (0.05 mg/kg, IV, Dolorex, MSD Animal Health A/S, Denmark). General anaesthesia was induced using diazepam (0.5 mg/kg, IV, Diazedor Vet., Salfarm Danmark A/S, Denmark) and ketamine hydrochloride (2.5 mg/kg, IV, Ketador Vet., Salfarm Danmark A/S, Denmark). Inhalation anaesthesia was maintained with isoflurane (1.5%, Vetflurane, Virbac Danmark A/S, Denmark). Tetanus antitoxin (1,500 units/animal SC, Tetanus antitoxin Equine Origin, Colorado Serum Company, Colorado) and antibiotics were administered prior to surgery. Antibiotic therapy included gentamicin sulphate (6.6 mg/kg BW, IV, Gentavet, ScanVet Animal Health A/S, Denmark) and benzylpenicillin sodium (22,500 IU/kg BW, IV, Benzylpenicillin, Panpharma Nordic AS, Norway), with the latter re-dosed every 80 min during surgery. Intraoperative analgesia consisted of flunixin meglumine (2.2 mg/kg BW, IV, Wellicox, Ceva Animal Health A/S, Denmark), and a SC ring block on each forelimb using bupivacaine hydrochloride (0.1 mg/kg BW, SC, Bupivacaine Baxter, Baxter A/S, Denmark) and mepivacaine hydrochloride (0.4 mg/kg BW, SC, Mepidor Vet, Salfarm Danmark A/S, Denmark).

### 2.4 Surgical protocol

The sheep were placed in dorsal recumbency with flexed, stabilised forelimbs. A tourniquet was applied mid-metacarpally. A dorsolateral curved incision was made, and the bone was exposed by blunt dissection and periosteal elevation. The extensor and flexor tendons were retracted and protected. In the ostectomy group removal of the bone section was conducted by first approximating the middle of the phalanges from an intraoperative radiograph. Then a conical 0.6 mm sawblade (DePuy Synthes) was applied to cut the bone halfway through, after which a custom made 3- or 6-mm cutting guide was inserted to guide the saw blade and make a standardised ostectomy size (Figure 1A; Supplementary Figures 1a,b). Each sheep in the ostectomy group received both a 3 mm and a 6 mm ostectomy, randomly assigned to either the left or right front limb proximal phalanx (Table 1).

**Table 1:** Overview of the population, fracture model, osteosynthesis method, aftercare, and bone healing parameters.

Sheep ID (limb) and group	Osteosynthesis	Aftercare		Implant stability (week no.)*	Alignment (degrees)	Time of first signs of callus formation in the gap	Healing status at week 16	Callus thickness (mm)
		Block	Cast/bandage					
P1 (LF) Ostectomy 3 mm gap	6-hole locking plate, unicortical screws	Week 0–10	Bandage week 0–8	Screw loosening (1, 3, 10); plate dislodgement (10)	6.8	No callus	No bone formation in gap; rounding of fracture edges	NA
P2 (RF) Ostectomy 3 mm gap	6-hole locking plate, unicortical screws	Week 0–10	Bandage week 0–8	Screw loosening (10, 14); plate dislodgement (14)	6.3	Week 12	Moderate bone formation in gap	1.55
P3 (RF) Ostectomy 3 mm gap	6-hole locking plate, unicortical screws	Week 0–10	Bandage week 0–8	Screw loosening (1); screw breakage (4, 6); plate dislodgement (6)	7.6	Week 8	Moderate bone formation in gap	1.3
P4 (LF) Ostectomy 3 mm gap	6-hole locking plate, unicortical screws	Week 0–10	Bandage week 0–8	Screw breakage (8, 12)	0.2	Week 10	Moderate bone formation in gap	2.55
P1 (RF) Ostectomy 6 mm gap	6-hole locking plate, unicortical screws	Week 0–10	Bandage week 0–8	Screw breakage (8, 16); plate dislodgement (16)	11.5	No callus	No bone formation in gap; rounding of fracture edges	NA
P2 (LF) Ostectomy 6 mm gap	6-hole locking plate, unicortical screws	Week 0–10	Bandage week 0–8	Plate dislodgement (10)	4.5	No callus	No bone formation in gap; rounding of fracture edges	1.9
P3 (LF) Ostectomy 6 mm gap	6-hole locking plate, unicortical screws	Week 0–10	Bandage week 0–8	Screw loosening (8); screw breakage (14)	3.1	No callus	No bone formation in gap; rounding of fracture edges	0.85
P4 (RF) Ostectomy 6 mm gap	6-hole locking plate, unicortical screws	Week 0–10	Bandage weeks 0–8	Screw loosening (4); screw breakage (8, 16)	0.3	No callus	No bone formation in gap; rounding of fracture edges	2.175
A1 (LF) Osteotomy Subgroup A	5-hole locking plate, unicortical screws	Week 6–10	Cast week 0–6, bandage week 6–8	Screw loosening (4)	6	Week 8	Clearly visible fracture line	2.95
A2 (RF) Osteotomy Subgroup A	5-hole locking plate, unicortical screws	Week 3–10	Cast week 0–3, bandage week 3–8	Intact	0.3	Week 8	Barely visible fracture line	2.08
A3 (RF) Osteotomy Subgroup A	5-hole locking plate, unicortical screws	Week 4–16	Cast week 0–4, bandage week 4–8	Screw loosening (1)	4.7	Week 12	Clearly visible fracture line	1.28
B1 (LF) Osteotomy Subgroup B	5-hole locking plate, bicortical screws	Week 0–10	Bandage week 0–8	Intact	2	Week 6	Barely visible fracture line	0.785
B2 (RF) Osteotomy Subgroup B	5-hole locking plate, bicortical screws	Week 0–10	Bandage week 0–8	Screw breakage (6, 12, 14)	4.6	Week 6	Clearly visible fracture line	2.35
B3 (RF) Osteotomy Subgroup B	5-hole locking plate, bicortical screws	Week 0–10	Bandage week 0–8	Screw breakage (6)	1.6	Week 6	Clearly visible fracture line, week 10**	NA
B4 (LF) Osteotomy Subgroup B	5-hole locking plate, bicortical screws	Week 0–10	Bandage week 0–8	Intact	0.5	Week 6	Barely visible fracture line	0.525

*Implant stability* is noted as either *screw loosening/breakage*, *plate dislodgement* or *intact*. *Alignment* denotes the difference in angulation between proximal and distal fragments, evaluated on radiographs from day 0 and week 12. *The first signs of callus formation* marks the week in which callus was first observed. *Healing status at week 16* is divided into *no* or *moderate bone formation in the gap* and registration of *rounding of fracture edges* (ostectomies), and *clearly* or *barely visible fracture lines* (osteotomies). Finally, *callus thickness* recorded from post-euthanasia CT scans is reported. RF, right front limb; LF, left front limbs. *Radiographs representing these events are depicted in Supplementary Figure 4. **Euthanized on week 10 due to hoof trauma.

In the osteotomy group (*n* = 7) a dorsal midshaft 4.5 mm unicortical defect was created using a 4.5 mm drill bit, followed by a midline osteotomy performed with a conical 0.6 mm sawblade (46/25 × 6 × 0.6/0.4 mm (519.230S), DePuy Synthes, Johnson & Johnson MedTech, Massachusetts, USA). After ostectomy or osteotomy, metal implant fixation was performed (Table 1).

The 3- and 6-mm ostectomies were fixated with a 6-hole cuttable stainless-steel locking plate (1.5 mm, Straight (02.114.005S), DePuy Synthes, Johnson & Johnson MedTech, Massachusetts, USA) using four unicortical locking screws (cortex stardrive, Ø 1.5 mm, L 10 mm (02.214.110S), DePuy Synthes, Johnson & Johnson MedTech, Massachusetts, USA), two in each fragment (Figure 1A). The osteotomies were fixated with a five-hole cuttable stainless-steel locking plate (shorter version of plate used for ostectomies) with either four unicortical screws (*n* = 3 phalanges, subgroup A, same screw types as described for ostectomies) or four bicortical screws (*n* = 4 phalanges, subgroup B, Ø 1.5 mm, L 16 mm (VS106.018), DePuy Synthes, Johnson & Johnson MedTech, Massachusetts, USA) (Figure 1B; Supplementary Figure 1c,d).

After the osteosyntheses were completed, the soft tissues were closed by simple continuous pattern (fascia) (Novosyn 2/0, B Braun Medical Inc., USA) and simple interrupted pattern (skin) (Optilene 2/0, B Braun Medical Inc., USA).

### 2.5 Post-surgical coaptation

To find the most useful coaptation, two different approaches were adopted. In the osteotomy sheep (*n* = 4) a hoof block adjusted to fit an ovine digit was applied. This had a height of 20 mm (Hoof block, standard, Klovshoppen, Denmark), and it was glued (Mini Moo Gloo, MooGloo, Durham, USA) under the medial digit on both front limbs for offloading of the osteosynthesized digit. Additionally, a supportive and protective elastic bandage was applied over the surgical site (Figure 1C). In osteotomy subgroup A (*n* = 3 sheep), a half-limb walking cast was applied for approximately 4 weeks post-surgery (Figure 1C), hereafter elastic bandages and hoof blocks were applied until week 8 (Figure 1C). In subgroup B (*n* = 4 sheep), we opted to use hoof block offloading and bandages as described for the ostectomy group. All sheep were permitted to ambulate unrestrictedly post-surgery. In all sheep, the elastic bandages were removed at week 8 and the hoof blocks were removed at week 10 after surgery. In the osteotomy group, one sheep was euthanized on day 70 due to hoof trauma.

### 2.6 Radiography and computed tomography

Radiographs were obtained throughout the 16-week study period, i.e., intra-operatively and at 1, 2, 4, 6, 8, 10, 12, 14, and 16 weeks. For the post-surgery radiography, the sheep were lightly sedated with xylazine (0.08 mg/kg BW, IV, Xysol Vet., ScanVet Animal Health A/S, Denmark) and butorphanol tartrate (0.04 mg/kg BW, IV, Dolorex, MSD Animal Health A/S, Denmark). At week 16, radiography and CT were acquired immediately after euthanasia. Each radiographic study entailed three projections: dorsopalmar, dorsomedial-palmerolateral oblique, and lateromedial. A portable Gierth TR80/20 system was used, set to 60 kV and 3 mAs, along with Fujifilm FDR D-EVO console software. For CT, a Siemens Somatom Emotion (46,213, 14o kV) was used.

From the radiographs, the following parameters were recorded to investigate fracture fixation stability and bone healing:

1. *Implant component stability* was classified based on occurrences of screw loosening and/or plate dislodgment.
2. *Fragment alignment* was defined as the difference in angle degrees between the proximal and distal fragment in anterior–posterior projection at the intraoperative radiographs vs. radiographs at week 12. Week 12 was chosen to allow for an evaluation of the effect of removing the offloading hoof-block in relation to fragment alignment, which was performed at week 10.
3. *First signs of callus formation* in the fracture gap were recorded and the corresponding week was noted (Table 1).
4. *Healing status at week 16* was categorised by using parameters from two individual healing scoring systems: radiographic union scale in tibial fractures (RUST) and Lane and Sandhu scoring system (36, 37). The RUST system scores each cortex (anterior, posterior, medial, and lateral) based on two criteria: presence of callus and visibility of the fracture line. A cortex receives 1 point if no callus is present and the fracture line is visible, 2 points if callus is present but the fracture line remains visible, and 3 points if callus is present and the fracture line is no longer visible. The Lane and Sandhu system evaluates bone healing based on three parameters: bone formation at the fracture gap (none, moderate, or complete), visibility of the fracture line (clearly visible, blurred, or not visible), and degree of remodelling (none, beginning signs, or complete remodelling). ‘*Presence of bone formation in the fracture gap*’ (Yes/No), was applied in the ostectomy group only, whilst ‘*fracture line visibility (clearly visible, blurred or not visible)*’ was applied in the osteotomy group. Clearly visible fracture lines were defined as uninterrupted and sharply delineated lines, corresponding to the lowest score in the Lane and Sandhu system. Additionally, the presence of rounding at the fracture edges was recorded, as an indication of potential non-healing (Table 1).

*Callus thickness* was measured from CT images, assessing the radiographically dense (mineralised) hard callus visible on the scans (Supplementary Figure 2). Callus thickness was measured in two designated areas on each fragment, yielding four measurements per sheep. The mean value was reported. The first measurement was taken at the subjectively identified thickest region of callus on transverse CT images. From this point, a line was drawn towards the centre of the bone, and a second line was drawn from the centre at a 45° angle relative to the first line, towards the cortex, directed away from the plate. Callus thickness was then measured along this second line (Supplementary Figure 2).

### 2.7 Post-surgery care and welfare monitoring

Following surgery, all sheep received antimicrobial and analgesic treatment. Gentamicin sulphate was administered once daily (6.6 mg/kg, IV, Gentavet, ScanVet Animal Health A/S, Denmark), and benzylpenicillin sodium was given intravenously every 6 h (22,500 IU/kg, IV, Benzylpenicillin, Panpharma Nordic AS, Denmark) – the ostectomy groups received antimicrobial treatment for a duration of 7 days, this was downregulated for the osteotomy group for a duration of 3 days.

For pain management, flunixin meglumine (2.2 mg/kg, IV; Wellicox, Ceva Animal Health A/S, Denmark) was administered twice daily for the first 3 days, followed by once-daily dosing until day eight.

The sheep were closely monitored throughout the study period. Throughout the study period they were observed by technical staff twice daily, where appetite, general well-being and intactness of the coaptation was assessed. During the first 2 weeks after surgery, clinical assessments were conducted by veterinarians two to five times daily, with frequency decreasing over time. In weeks 3–5 veterinary assessment occurred twice a week, and for the rest of the study period once a week. The clinical assessment included temperature (by scanning the temperature-sensitive chip), heart rate, respiratory rate, rumen contractions, lameness and two pain scores. The weight of the animals and their body condition score were evaluated several times during the study period.

The hoof block was continuously monitored and adjusted as needed to optimise the sheep’s ambulation, including increasing the weight-bearing surface by adding composite material on the edges of the hoof block (Supplementary Figure 3). A detached hoof block was immediately replaced. Adjustments or replacements were performed under light sedation, as described above.

### 2.8 The sheep pain facial expression scale (SPFES)

Pain facial expressions were assessed using the SPFES developed by McLennan et al. (2016) (38). Five facial regions were scored based on the presence of pain-related expressions: (1) tightening of muscles surrounding eyes, closing of the eye, (2) tightening of the muscles of the cheek and in area of the zygomatic arch, (3) ear position, (4) lip and jaw profile, and (5) shape of nostrils and philtrum. The sheep were observed for 2 min from a distance to avoid interference with their potential pain-related facial expression. The SPFES has a maximum total score of ten. Three levels of treatment criteria were defined based on the SPFES (Table 2).

**Table 2:** Treatment criteria applied for total scores of the sheep pain facial expression scale (SPFES) and for total scores of the ovine orthopaedic pain behaviour score (OOPBS).

Total score, SPFES	Total score, OOPBS	Action
≤3	<7	No action
4–5	7–12 or lameness 3, 4 or on knees 3	Treatment criterion 1: Analgesic medication changed/increased, increased monitoring
≥6	>12	Treatment criterion 2: Analgesic medication changed/increased – if no effect, euthanasia

SPFES: sheep pain facial expression scale [maximum total score of 10 as described in McLennan et al. (38)]. OOPBS, ovine orthopaedic pain behaviour score (maximum score of 23).

### 2.9 The ovine orthopaedic pain behaviour score (OOPBS)

Initially, the clinical severity score (CSS) described by Häger et al. (21) was utilised for the ostectomy group. The CSS includes assessments of food/water consumption, lameness, activity and presence of vocalisation. Based on the CSS and experience from the ostectomy group, the novel *a priori* weighted composite OOPBS was developed to create a more refined and sensitive pain scoring system, tailored to the behavioural signs relevant in orthopaedic research. By incorporating specific indicators such as posture and general demeanour, the new *a priori* weighted composite OOPBS aimed to provide a more sensitive and accurate assessment of pain. The OOPBS included assessment of appetite, general demeanour, vocalisation/teeth grinding, general activity, lameness, kneeling, and other adverse behaviours (such as flehmen and wool picking) (Table 3). The sheep were observed for 2 min from a distance. The maximum attainable score was 23, with varying weights assigned to different parameters, reflecting their proposed significance, similar to what has been done in horses (39). Three levels of treatment criteria were defined (Table 2).

**Table 3:** The ovine orthopaedic pain behaviour score (OOPBS).

Parameter	Description	Score
Feed*	Normal, interested, been eating	0
	Reduced interest	1
	Inappetence (no interest, not eating)	2
General demeanour	Bright, alert, responsive	0
	Quiet, alert, responsive	1
	Moderately depressed and inactive	3
	Depressed, inactive and nonresponsive	4
Vocalisation	None	0
	None, teeth grinding	1
	Occasionally vocalising or teeth grinding	2
	Frequently vocalising or teeth grinding	4
Activity	Normal, resting and sleeping	0
	Frequent change of position	1
	Restless	2
Lameness	Normal standing and walking	0
	Normal standing, minimal lameness in the walk	1
	Normal standing, lameness in the walk	2
	Relief of the affected leg, high lameness in the walk	3
	No usage of the affected leg, predominantly in suspension	4
On knees	Not on knees	0
	Occasionally on knees, e.g., when eating	1
	Frequently on knees when eating and walking	2
	Constantly on knees	3
Other	None	0, 0
	Flehmen	2
	Wool picking	2
**Maximum score**		**23**

*Interested/eating hay and/or pellets or have been eating (reduced amount of hay in the stall), rumination observed. Modified from Häger et al. (21).

If a sheep reached a treatment criterion in either the OOPBS and/or the SPFES, increased monitoring was initiated, and actions such as adaptation of the coaptation and/or additional analgesic treatment were taken. Available options for supplementary analgesia included flunixin meglumine (2.2 mg/kg, IV, Wellicox, Ceva Animal Health A/S, Denmark), butorphanol tartrate (0.2 mg/kg, IM, Dolorex, MSD Animal Health A/S, Denmark), meloxicam (1 mg/kg, SC, Metacam, Boehringer Ingelheim Vetmedica GmbH, Germany), transdermal fentanyl patches (100 μg/h transdermal, Fentanyl Sandoz, Sandoz A/S, Denmark), buprenorphine hydrochloride (0.01 mg/kg, IM, Bupaq Multidose Vet., Salfarm Danmark A/S, Denmark), or regional nerve blocks mid-metacarpal using bupivacaine hydrochloride (0.3 mg/kg BW, SC, Bupivacaine Baxter, Baxter A/S, Denmark) and mepivacaine hydrochloride (1.0 mg/kg BW, SC, Mepidor Vet, Salfarm Danmark A/S, Denmark) (40).

### 2.10 Biomarkers

Blood and saliva samples were collected preoperatively (day −1) and on days 2, 4, 6, 7, and 14, and hereafter week 3, 4, 6, 10, and 14. All samples were collected at the same time a day, to avoid diurnal influences.

Prior to saliva collection, the sheep’s mouth was rinsed with 30 mL of tap water using a syringe. Saliva samples were collected using Salivette® tubes (Sarstedt Aps, Bording, Denmark), which consists of an absorbent component and a centrifuge tube designed for non-invasive saliva collection. The original absorbent component in the Salivette® was replaced with sponges (Esponja Marina, La Griega E. Koronis, Madrid, Spain). A sponge was held with forceps inside the sheep’s mouth for 30–60 s. The saliva-saturated sponge was transferred to a Salivette® tube. This procedure was repeated with one or two additional sponges until an adequate volume of saliva was collected. The Salivette® tube was centrifuged within 30 min after collection at 3000 × g for 10 min at 4 °C and saliva transferred to a cryotube (Maxxline Cryo Tubes, non-sterile, Dacos, Hvidovre, Denmark). Collecting saliva was a non-invasive and cheap procedure to which the sheep quickly adapted.

Blood was collected by an indwelling intravenous catheter (Long-Term MILACATH® – Large Animal Kits – Seldinger Technique, Mila International, Inc., Kentucky, USA) or by venipuncture of *v. jugularis*. The blood samples were collected in BD Vacutainer® 4 mL tubes coated with lithium heparin (Becton Dickinson Vacutainer Systems Europe) for plasma. The samples were centrifuged within 30 min of collection at 1,800 × g for 10 min at 4 °C and plasma transferred to cryotubes (Maxxline Cryo Tubes, Dacos, Hvidovre, Denmark).

All samples were stored at −80 °C until analysis. Cortisol levels in saliva were measured by alphaLISA technology according to López-Arjona et al. (41). Serum amyloid A (SAA) and haptoglobin concentrations were obtained with commercially available kits as described by Franco-Martínez et al. (42) (VETSAA; EIKEN Chemical Co., LTD; Tokyo, Japan) and Schmidt et al. (43) (kit haptoglobin Tridelta phase range, Tridelta Development Ltd., Bray, Ireland), respectively. The serum kits showed an intra- and inter-assay imprecision lower than 15% and were linear (*r* > 0.91) after serial sample dilutions.

### 2.11 Digital circumference

The circumference of the proximal phalanges was measured using a string, which was placed at the height of the distal aspect coronary band of the dewclaws. The length of the string was then measured with a ruler to obtain the circumference. The same investigator undertook the measurements each time. Circumference was measured regularly. The first measurement was taken on the day of surgery and at regular intervals post-surgery, except in osteotomy subgroup A, where the initial casting prohibited assessment of circumference.

### 2.12 Statistics

Descriptive statistics was performed by applying Excel® (Microsoft Excel software version 2,504, Microsoft Corporation, Redmond, WA, USA) and GraphPad Prism (version 10.4.2 for Windows, GraphPad Software, Boston, Massachusetts USA).^1^

## 3 Results

Results are reported for implant integration and fracture fixation stability, as well as healing progression, assessed through imaging techniques (radiographs and CT). Clinical assessments, pain evaluation via facial expression and behavioural scoring systems, as well as biomarkers of stress and inflammation, were applied to provide a comprehensive evaluation of treatment efficacy and animal welfare.

### 3.1 Imaging assessment of fracture fixation stability and healing

#### 3.1.1 Radiographic evaluation

*Implant stability* (Table 1; Supplementary Figures 4a–d): In the ostectomy 3 mm gaps, four out of four phalanges had screw loosening or breakage, and two experienced plate dislodgement. In the ostectomy 6 mm gaps, all four phalanges had screw breakage or plate dislodgement at varying time points, starting from week 4. In the osteotomies, plate dislodgment was not observed. Screw loosening or breakage occurred in 2 out of 3 phalanges in subgroup A and 2 out 4 phalanges in subgroup B.

*Healing status (week 16)* (Table 1; Supplementary Figures 4e–h): At week 16, three of the four digits with 3 mm gaps showed moderate bone formation, with the last showing no bridging and rounding of the fracture edges. In the digits with 6 mm gaps, none had bone formation within the gap, and all had evident rounding at the fracture edges, indicating potential non-union. In the osteotomy subgroup A, two digits retained clearly visible fracture lines, whilst one showed a barely visible line. In the osteotomy subgroup B, 2 phalanges had barely visible fracture lines, the other two had osteotomy lines that remained clearly visible.

*Alignment* (Table 1): In the 3 mm ostectomies, the mean change in alignment from intraoperative measurements to week 12 was 5.2° (range 0.2–7.6°) whilst the 6 mm ostectomies showed a mean change of 4.9° (0.3–11.5°). Osteotomy subgroup A and B had an average change of 3.7° (0.3–6.0) and 2.2° (0.5–4.6°), respectively.

*First signs of callus* (Table 1): In the ostectomy 6 mm gaps, no callus formation across the fracture gap was observed within the study period. In the ostectomy 3 mm gaps, however, callus formation was observed in 3 out 4 sheep, at week 8, 10 and 12, respectively. In contrast, in the osteotomy subgroup A and B, callus formation across the fracture area was observed in all cases, after a mean time of 9.3 weeks and 6 weeks in the subgroup A subgroup B, respectively.

#### 3.1.2 Computed tomography evaluation

*Callus thickness* (Table 1): A mean callus thickness of 1.8 mm (range 1.3–2.55 mm) and 1.6 mm (range 0.85–2.18 mm) was found in the 3- and 6-mm ostectomies that showed new bone formation, respectively. In the osteotomy subgroups A and B, mean callus thickness was 2.1 mm (1.28–2.95 mm) and 1.2 mm (0.53–2.35 mm), respectively.

### 3.2 Clinical monitoring

#### 3.2.1 First week after surgery

Within the first week after surgery (days 1–7), the majority of the 11 included sheep were classified as bright, alert, and responsive (130 observations out of a total of 152). The sheep generally had a good appetite during this period (143 observations of good/normal appetite out of 152). One sheep (A3) stood out as more affected after surgery with diarrhoea (days 1–8), pigmented urine (days 1 to 3), decreased general demeanour (days 1 and 2), and decreased appetite (days 1–3), all of which responded to treatment within the first week. Within the first week, six of the 11 sheep exhibited soft or watery faeces, which resolved within one to 4 days.

Within the first week after surgery, two sheep had body temperature >40 °C twice. The temperature returned to the normal range [39–40 °C, (44)] after 12–24 h and remained with reference level for the duration of the study in all sheep. Within this week, the mean heart rate was 77 beats per minute [with a range of 44 to 120 beats per minute, normal range: 70–80 (44)]. Ten sheep had heart rate >84 beats per minute (75% percentile) 1–5 times within the first week after surgery. Mean respiratory rate was 41 breaths per minute [with a range of 16–84 breaths per minute, normal range: 12–20 (44)]. Six of the sheep had a respiratory rate of >60 breaths per minute (75% percentile), measured between one and four times during this one-week period.

#### 3.2.2 Body condition score and weight changes during the study period

According to the AWIN welfare assessment protocol for sheep (33), the body condition score of all eleven sheep ranged from 2.0 to 4.0 in the classification “Good,” throughout the study period (33). The weight changed from −4.5 kg to +3.5 kg from the starting weight. However, there were no significant difference from start to final body weight (paired *t*-test, *p* = 0.78).

#### 3.2.3 Hoof block maintenance

The average wear life of the hoof blocks was 25 days (median: 23.5 days, interquartile range: 12–39 days), at which point they spontaneously detached from the hooves and had to be replaced.

#### 3.2.4 Pain scores

**Figure 2:**
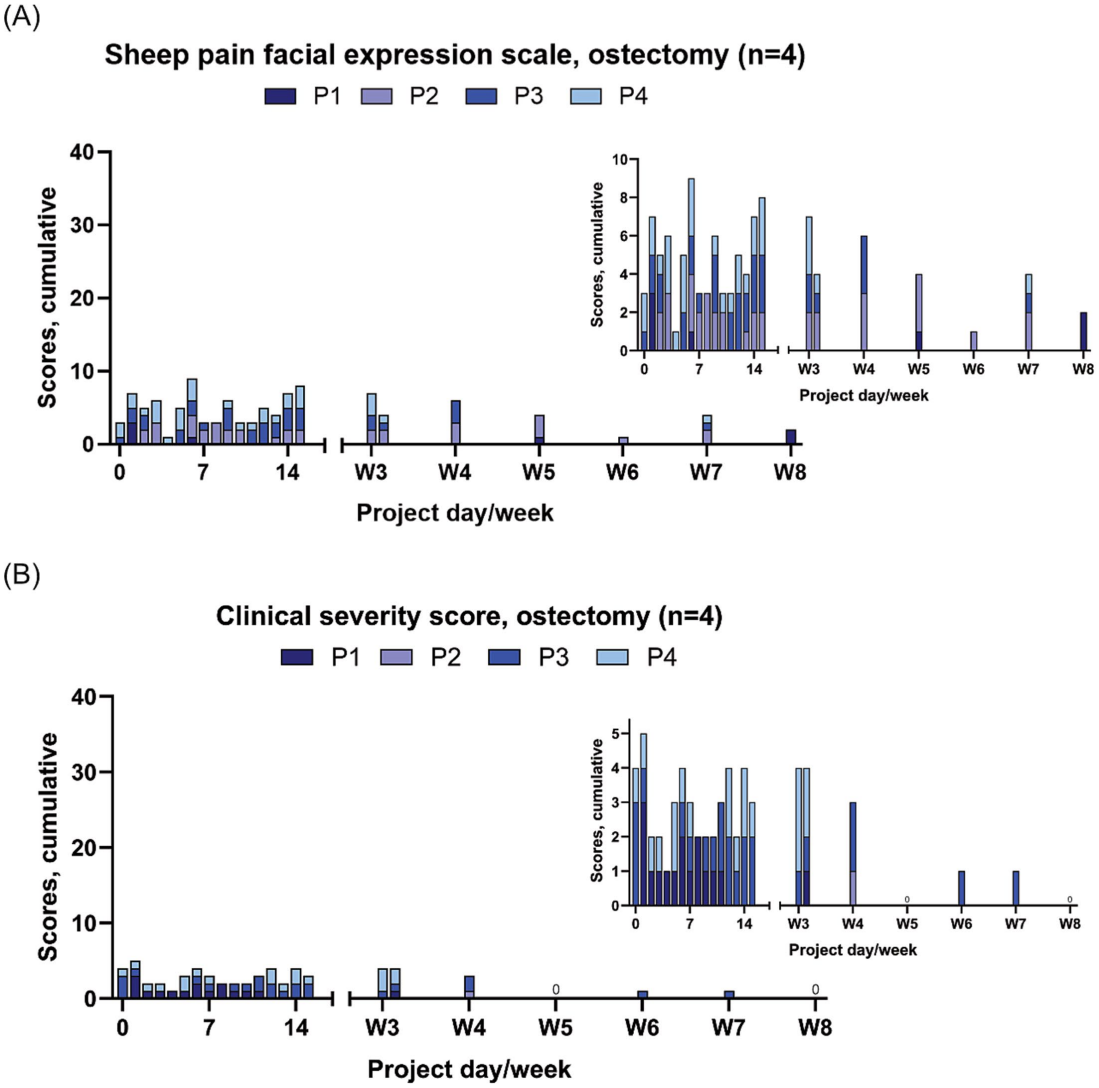
Pain score results from the sheep with ostectomies (*n* = 4). The results are presented as cumulative scores, with the y-axis representing the highest attainable score (4 x the maximum score), insert graphs visualising results in detail. Note that these graphs represent only 4 animals (important for comparison with cumulative data from 7 sheep in Figure 3). **(A)** Sheep pain facial expression scale [SPFES; (38)] and **(B)** clinical severity score [CCS; (21)]. 0: All sheep scored 0.

**Figure 3:**
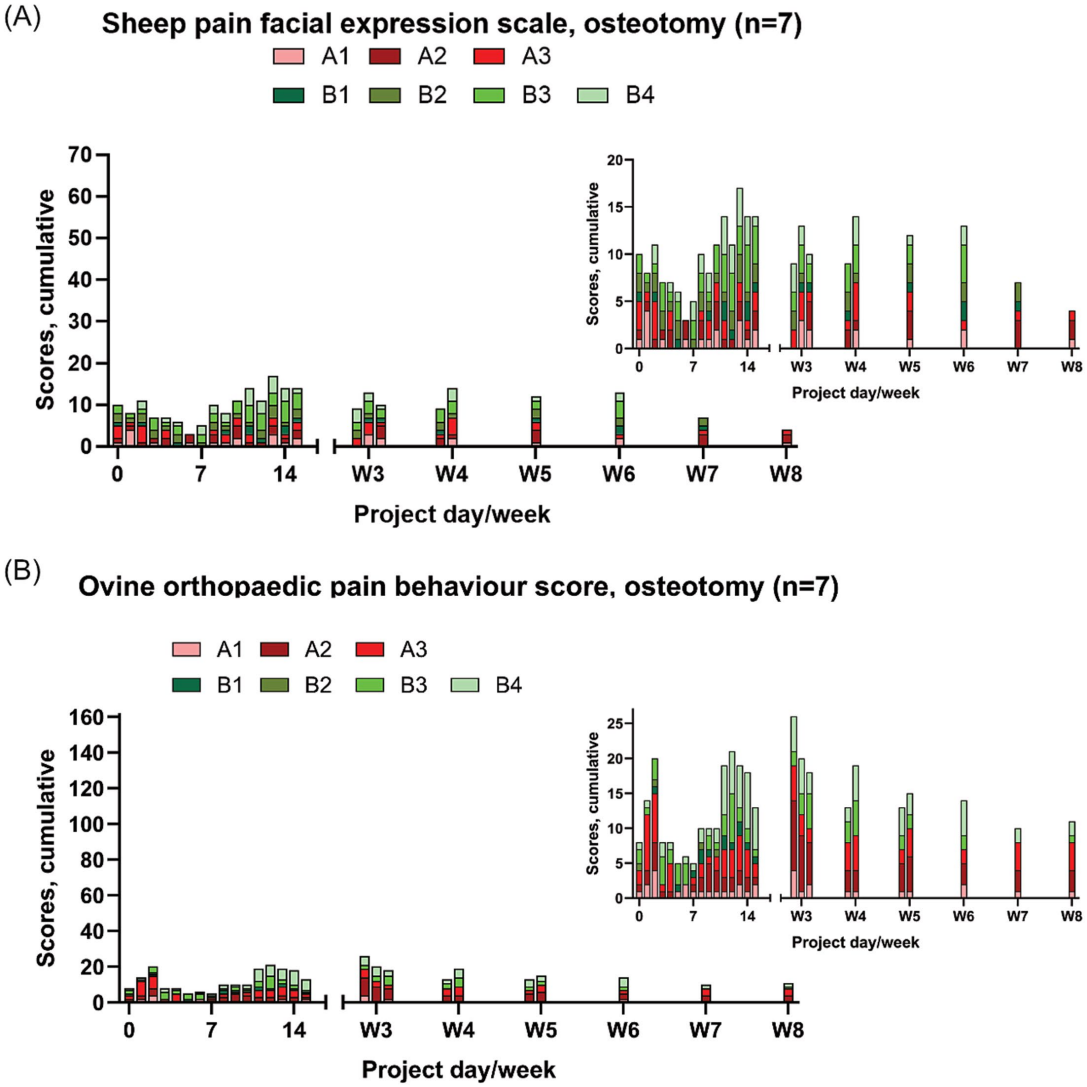
Pain score results from the sheep with osteotomies (*n* = 7). The results are presented as cumulative scores, with the y-axis representing the highest attainable score (7 x the maximum score), insert graphs visualising results in detail. Note that this graph represent data from 7 sheep (in comparison to the 4 sheep in Figure 2) **(A)** sheep pain facial expression scale [SPFES; (38)] and **(B)** the ovine orthopaedic pain behaviour score (OOPBS).

Throughout the study period, the sheep in the ostectomy group individually scored from 0 to 3 on the CSS and individually scored from 0–3 on the SPFES (Figure 2). The sheep included in the osteotomy group individually scored from 0 to 8 on the OOPBS and individually scored from 0 to 5 on the SPFES (Figure 3).

The pain scores (CSS, OOPBS, SPFES) exhibited similar patterns of change over the study period, with an initial peak on days 1–2 post-surgery, then a gradual decline, and a second peak appearing 2–3 weeks after surgery (Figures 2, 3). The OOPBS provided a more detailed scoring system than the CSS.

From week eight until the end of the study period there were minimal pain scores. Occasionally a sheep scored one or two on the SPFES but with no apparent pattern (data not shown).

#### 3.2.5 Lameness

**Figure 4:**
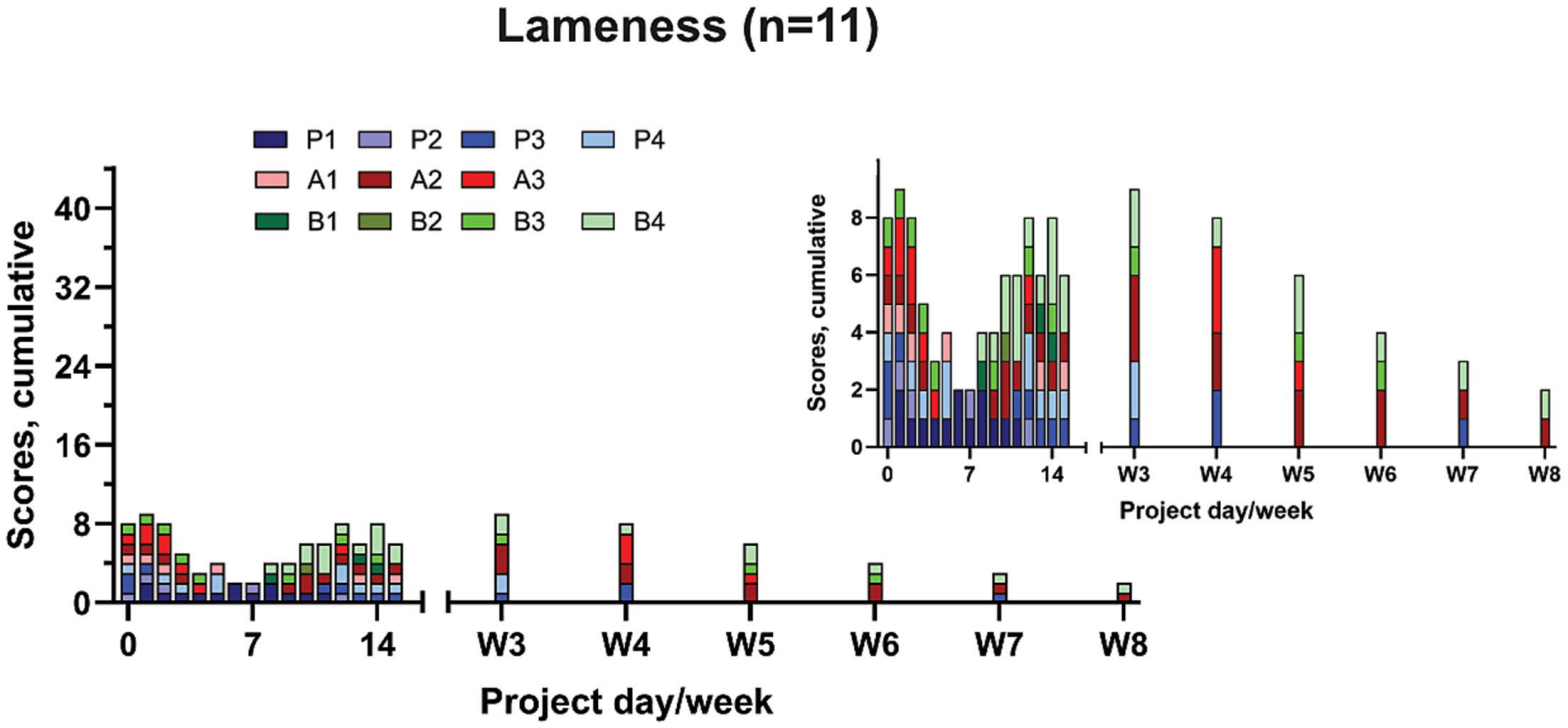
Lameness scores extracted from the clinical severity score from the sheep with ostectomies (*n* = 4) and the ovine orthopaedic pain behaviour score from the sheep with osteotomies (*n* = 7). The results are presented as cumulative scores, with the y-axis representing the highest attainable score, insert graphs visualising the results in detail.

Lameness scores were extracted from the CSS and OOPBS (Figure 4). Only one (A3) of the eleven sheep scored 3 (Figure 4); apart from this one case, the maximum lameness score was two. After week four, the lameness score declined.

#### 3.2.6 Treatment criteria

In the ostectomy group, SPFES treatment criterion 1, defined as a total SPFES score of 4–5 (Table 2), was reached 3 out of 448 days (4 × 112 days). In the osteotomy group, SPFES and/or OOPBS treatment criterion 1, defined as either a total SPFES sore of 4–5, a total OOPBS score of 7–12, a lameness score of 3–4, or an ‘on knees’ score of 3 (Table 2), was reached on 32 out of 742 days ((6 × 112) + (1 × 70) days). When a sheep reached treatment criterion 1, increased monitoring was initiated, and actions such as adaptation of the coaptation and/or additional analgesic treatment were taken. No sheep reached treatment criterion 2 during the study period (Table 2).

### 3.3 Biomarkers

#### 3.3.1 Cortisol

**Figure 5:**
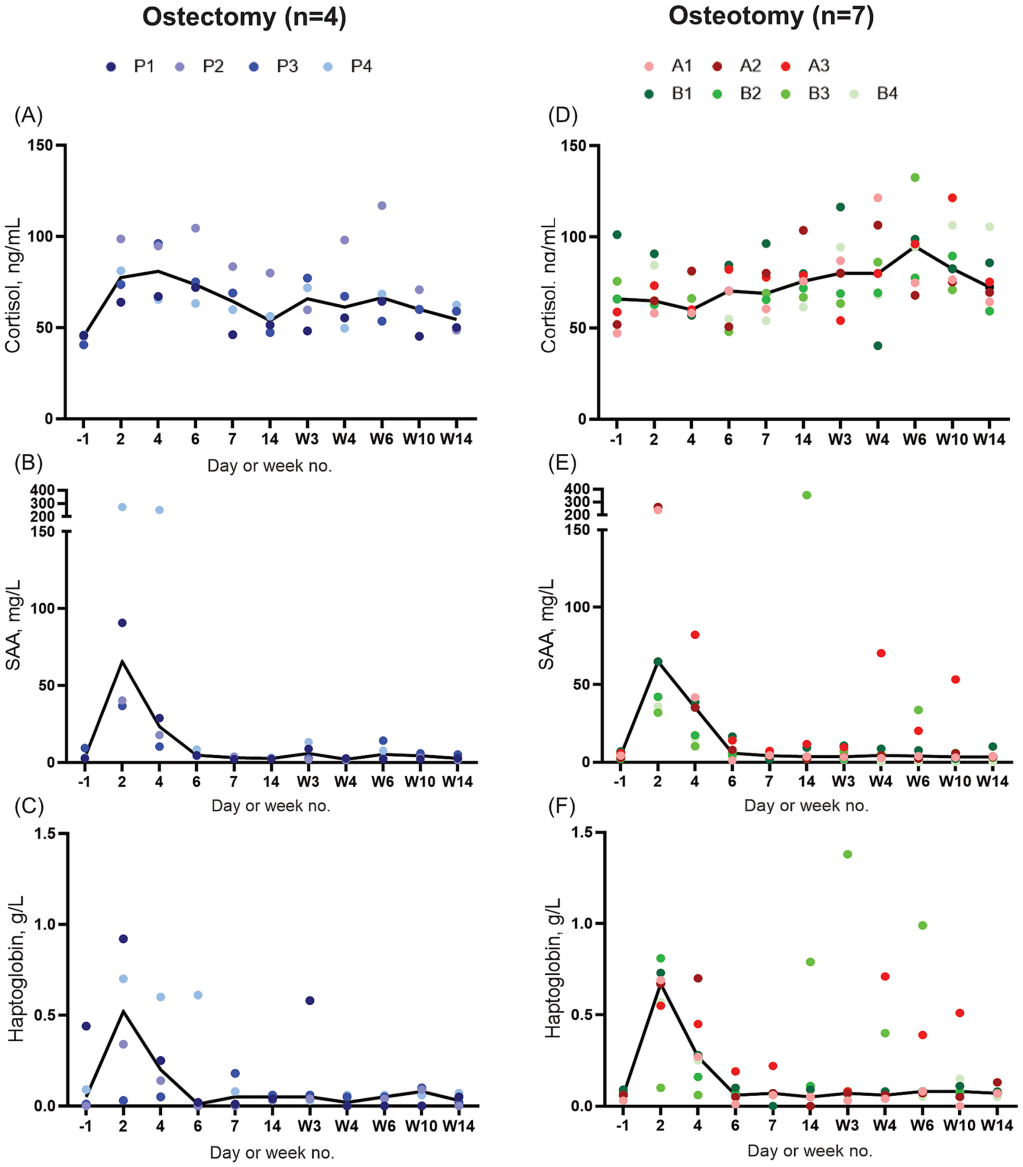
Biomarkers measured from the sheep (*n* = 4) included in the ostectomy group **(A–C)** and from the sheep (*n* = 7) included in the osteotomy group **(D–F)**. Saliva cortisol **(A,D)** and plasma SSA **(B,E)** and haptoglobin **(C,F)**. Solid line: median. SAA, Serum amyloid A.

In both groups, saliva cortisol baseline levels from the days prior to surgery ranged from 40.54 to 101.59 ng/mL with seemingly higher levels in osteotomy subgroup B (65.9–101.6 ng/mL) (Figures 5A,D). In the ostectomy group, cortisol levels peaked within the first week post-surgery and then declined gradually towards baseline (Figure 5A). In the osteotomy group, cortisol levels increased more slowly, with a peak at week 6, before gradually decreasing (Figure 5D).

#### 3.3.2 Acute phase proteins

The acute phase proteins SAA and haptoglobin exhibited an initial peak within the first few days post-surgery (Figures 5B,C,E,F), consistent with post-surgical inflammatory response. Thereafter, median levels remained low throughout the study period. In the osteotomy group, outliers were primarily represented by two animals, A3 and B3. These occasional concentration increases coincided with a bout of fever of unknow origin, lameness from loosening of hoof blocks/poorly fitting bandages, and a loose screw.

### 3.4 Circumference

Post-surgery, the circumference of the phalanges peaked at week 2 in the ostectomy group, followed by a decrease. From week 8 onwards, no consistent change in circumference was observed in this group (Supplementary Figures 5a,b). No obvious differences in circumference were identified between the 3- and 6- mm ostectomies. There were no consistent changes in circumference in the osteotomy group (Supplementary Figure 5).

## 4 Discussion

To our knowledge, this is the first large-animal fracture model specifically designed for assessing healing in small tubular bones in freely ambulating sheep. It enables the evaluation of fracture fixation and defect reconstruction strategies in a translational context, using a novel coaptation method that avoids restrictive slings to support animal welfare. The model was surgically feasible, allowed the sheep to move unrestrictedly after surgery, and enabled within animal comparisons. Implant failures occurred, demonstrating inadequate fracture fixation. However, when comparing implant stability, alignment, callus formation, and healing status at week 16, the osteotomy group showed signs of healing despite the mechanical instability. In contrast, the ostectomies were predominantly unstable, particularly the 6 mm ostectomies. Whilst the 3 mm ostectomies demonstrated moderate healing in three out of four phalanges, the 6 mm ostectomies consistently demonstrated non-healing with no callus bridging the defect. The consistent indications of non-union suggests a critical-size defect. None of the combinations of surgical approaches and coaptation methods ensured reliable healing, and these need to be refined to achieve a model with consistent healing outcomes. Use of sturdier implants and/or double plating could potentially yield more stable fixation and should be explored in future studies. Failure of repair is a common complication in large animal fracture models, with other studies main complication reported being implant failure/failure of fixation/osteotomy instability, additional one study reports spiral fracture in relation to the metal implant (16–18, 21, 24, 25, 30, 45, 46).

The overall welfare outcomes of this study were favourable, with low pain scores, limited stress responses, and acceptable recovery. In the light of increasing societal and scientific concerns regarding ethics, animal welfare, and validity of fracture research involving sheep and other experimental animals, we propose that phalangeal ostectomy and/or osteotomy could become a future welfare-oriented large animal fracture model. With further development and validation of this novel fracture model, it can provide a platform to investigate novel fracture fixation and bone healing materials and techniques to optimise treatment of small tubular bone fractures and defects.

The most effective coaptation method was not clearly identified. Offloading a diseased digit with a hoof block under the healthy digit to elevate the diseased digit is common in veterinary clinical practise in even-toed ungulates such as cattle, sheep, and goats (47) (Figure 1C; Supplementary Figure 3). In ostectomy subgroup B this led to healing in 2/4 phalanges and negated potential cast complications. However, maintaining hoof blocks proved time-consuming, as they were prone to detachment and required thorough surveillance accordingly.

Bone healing was evaluated using selected parameters from the RUST and Lane and Sandhu scoring systems, supplemented by CT-based callus measurements (36, 37). This approach was an attempt to standardise and quantify healing progression. However, due to implant failure—especially in the ostectomy 6 mm gap—bridging and callus formation were inconsistent and difficult to interpret. These scoring methods will become more applicable in future studies with enhanced mechanical stability in the model.

Surgical trauma, inflammation, pain and stress can cause changes in the biomarkers assessed in this study (48, 49). The cortisol levels peaking within the first weeks after surgery in the ostectomy group (a 1.8-fold peak on day 4, relative to baseline) might be a response to the surgical trauma. It is not clear why cortisol levels peaked later in the osteotomy group (a 1.4-fold peak in week 6, relative to baseline). The increase in cortisol experienced in the sheep in our study ranged between that found in sheep after minor stressors such as facing a dog (0.5-fold change), shearing (1.4 fold increase), or handling (1.6-fold increase) (50, 51) and major stressors such as orthopaedic surgery with tibial osteotomy (4-fold increase) (21) or mulesing, i.e., cutting crescent-shaped flaps of skin from around the breech and tail with sharp shears in the unanaesthetised animal (3-fold increase) (50).

The acute phase proteins, SAA and haptoglobin, increased rapidly in response to the surgical trauma (Figures 5B,C,E,F) (48, 49). Notably, the magnitude and pattern of this increase were comparable between the two groups, suggesting similar surgical trauma in the two groups. This is in contrast to our subjective impression that the surgical procedure in the ostectomy group was more invasive than in the osteotomy group. The phalangeal circumference increased more in the ostectomy group (Supplementary Figure 5), which could reflect the extent of surgical trauma, or a less stable osteosynthesis, with more oedema and haematoma formation the first weeks after surgery. Yet none of this was reflected in the concentrations of SAA and haptoglobin. Taken together, the biomarkers and phalangeal circumference suggest that the sheep experienced a minor-moderate surgical trauma.

The sheep recovered rapidly from anaesthesia and resumed eating within 30 min, additionally they able to ambulate freely after surgery. They had very limited expressions of pain, as this was scored as mild throughout the study period. Immediately after surgery, pain scores increased, peaked at day 2–3, and declined quickly again (Figures 2, 3). The mild pain was probably caused by the surgical trauma and/or discomfort related to post-fasting diarrhoea. A second peak in pain scores was observed around day 14 (Figures 2, 3). This may relate to the withdrawal of post-surgical NSAID treatment (administered until day 8) and underscores the importance of providing appropriate analgesia after surgery, even if it may affect bone healing (52). Use of pain scoring may thus assist in tailoring analgesic regimen post-surgically. Post-surgery pain management varies considerably in ovine orthopaedic models. One study used transdermal fentanyl for the entire duration of its 12-week study period (17), and other studies have used 10–13 days of analgesic treatments (NSAIDs, Fentanyl) (12, 21). However, the risk of gastric ulceration is a significant consideration when administering NSAIDs for a prolonged period. The majority of studies provide 3–5 days of analgesia, primarily NSAIDs and/or buprenorphine (16, 20, 23, 30, 45, 46, 53–55). It is not uncommon for studies to have no description of the analgesic treatment at all (22, 52–54). Our results suggest that 8 or more days of analgesia is needed, even with a smaller procedure such as the surgeries performed in our sheep.

Adopting strict treatment criteria (Table 2) ensured that the sheep received additional attention and pain medication if needed, and that no sheep experienced moderate or severe pain. Especially the *a priori* weighted composite OOPBS was of great benefit, as it entailed sensitive scoring system and treatment criteria (Tables 2, 3; Figures 2B, 3B), ensuring that even few expressions of pain prompted additional attention to the sheep and/or pain medication. Using an *a priori* weighted composite pain score such as the OOPBS offers the advantage of reduced subjectivity, as each category is clearly defined and pre-weighted. This enhances reproducibility and establishes the score as a valid tool for longitudinal pain assessment (56) and for comparison across studies or animals evaluated using the same scoring system.

For animal experiments to retain social licence to operate it is of the utmost importance that experimental animal welfare is high and pain is kept to a minimum and reporting is transparent (28) – and that there is appropriate reporting on these aspects of the experiment. In fracture and bone healing research there is an appalling paucity of reporting on the experimental animals’ clinical status, welfare, and pain manifestations. One fairly recent study on tibial osteotomy directly reports these data, including stress biomarkers, which were part of the study’s stated objectives (21), but otherwise welfare-related data are usually not described at all or only with few sentences (17–20, 45, 46, 53–55, 57–59). Some papers describe methods for pain assessment in the materials and methods section of the paper but fail to report the results (16, 25). A recent review of large animal models in articular cartilage repair similarly observed a void of information on pain management and monitoring in most published papers (26). Although the ARRIVE guidelines^2^ are available, there seems to be a need for journals reinforcing standardised and consistent reporting of pain and welfare of sheep and other large animals in orthopaedic research. Inadequate or incomplete reporting on experimental animal health and welfare is not only an ethical issue but may also lead to issues with reliability of the obtained data. Long-term stress, sustained pain, disease, or injuries can affect healing (60) and interpretation of the results, with potential negative impact on reliability and outcome measures of the study.

Pain recognition in animals through behavioural and facial expressions, has been a subject of scientific attention in several species for decades, building on foundational work by Darwin in 1872 (61). Thorough observation is necessary, as many prey animal species will do their utmost to hide pain, disease and weakness (62). Using facial expressions to recognise pain is common in veterinary practise and experimental animal research, and tools has been developed for numeral species (63). Methods for assessing welfare and pain in sheep have been reported and refined for several decades (21, 38, 56). The SPFES was validated by McLennan et al. (38) in sheep suffering from mastitis or footrot, and we found that the SPFES was easy to use and applicable in sheep in orthopaedic research. In future pain assessment of animals, artificial intelligence (AI) will most likely play a considerable role, as a recent study reports, that an AI tool significantly outperformed experienced veterinarians in recognising pain in sheep using the SPFES (38, 63).

Taken together, it is our impression that the OOPBS and the SPFES were easy to apply, and, when combined with the treatment criteria, they served as valuable clinical tools for assessing acute pain, documenting accumulated pain over time, gauging model severity, and for identification of animals in need of extra attention and/or pain medication. It is, however, important to acknowledge that confirmation bias may have affected the pain scores. Pain scoring was performed by direct observation of the sheep, and the principal investigators were therefore not blinded and were directly involved in implementing the therapeutic plan. Video-based pain scoring would minimise the influence by human presence on animal behaviour and allow for blinded assessments.

Conclusively, this study represents a first step towards developing a bilateral ovine proximal phalanx fracture and defect model with a comprehensive welfare monitoring plan. Ostectomy with 3–6 mm gaps lead to minimal or no healing, whilst osteotomy in combination with a 4.5 mm drill hole healed in some instances after repair with 1.5 mm 5-hole locking plates. To obtain consistent healing adjustments to the model are needed to fully accommodate the dynamic loading of the fracture site. However, the model concept is promising, and a modified model may provide translational insights into fracture healing and orthopaedic implant performance in smaller tubular bones. Importantly, pain scores remained low and the sheep moved freely in their pens, supporting the model’s feasibility from an animal welfare perspective. Moreover, the bilateral design allows paired comparisons, and from an experimental animal ethical standpoint it is desirable that the model allows reduced animal numbers and that welfare outcomes were favourable.

Additionally, with this study we challenge the current approach to animal welfare monitoring in sheep used in orthopaedic research. We have shown that qualitative and quantitative data on pain, stress, and welfare can and should be reported alongside parameters such as bone healing. Ensuring proper welfare monitoring and reporting are essential for both scientific integrity and societal acceptance of research involving animals.

## Data Availability

The raw data supporting the conclusions of this article will be made available upon reasonable request.

## Footnotes

^1^ www.graphpad.com

^2^ https://arriveguidelines.org/

## References

[1] Wu AM Bisignano C James SL Abady GG Abedi A Abu-Gharbieh E. Global, regional, and national burden of bone fractures in 204 countries and territories, 1990–2019: a systematic analysis from the global burden of disease study 2019. Lancet Healthy Longev. (2021) 2:e580–92. doi: 10.1016/S2666-7568(21)00172-0, PMID: 34723233 PMC8547262

[2] van Leerdam RH Krijnen P Panneman MJ Schipper IB. Incidence and treatment of hand and wrist injuries in Dutch emergency departments. Eur J Trauma Emerg Surg. (2022) 48:4327–32. doi: 10.1007/S00068-021-01732-X, PMID: 34196727 PMC9712291

[3] De Putter CE Selles RW Polinder S Panneman MJM Hovius SER Van Beeck EF. Economic impact of hand and wrist injuries: health-care costs and productivity costs in a population-based study. J Bone Joint Surg. (2012) 94:e56. doi: 10.2106/JBJS.K.00561, PMID: 22552678

[4] de Haas LEM van Lücht VAP Hoorn BT Salentijn DA Groenwold RHH Schep NWL. Patient-reported outcomes three months after treatment of metacarpal and phalangeal fractures or dislocations: a multicentre snapshot study. Bone Jt Open. (2025) 6:227–36. doi: 10.1302/2633-1462.62.BJO-2024-0146.R1, PMID: 40008527 PMC11863288

[5] Roennegaard AB Jensen SS Tengberg PT Gundtoft PH Viberg B. Risk of secondary surgery following surgical treatment of fractures: a nationwide register study on 9, 719 adult patients. Acta Orthop. (2025) 96:304–9. doi: 10.2340/17453674.2025.43446, PMID: 40223675 PMC11995429

[6] Wellborn PK Allen AD Draeger RW. Current outcomes and treatments of complex phalangeal and metacarpal fractures. Hand Clin. (2023) 39:251–63. doi: 10.1016/j.hcl.2023.02.002, PMID: 37453755

[7] Dargan D Wyman M Bhoora M Ronan D Baker M Partridge D. Hand osteomyelitis: a systematic review of the literature and recommendations for diagnosis and management. Hand. (2024). doi: 10.1177/15589447241284408, PMID: 39462293 PMC11559780

[8] Lazerges C Degeorge B Coulet B Chammas M. Diagnosis and treatment of hand tumors. Orthop Traumatol Surg Res. (2022) 108:103153. doi: 10.1016/J.OTSR.2021.103153, PMID: 34838755

[9] Liu ZB Liu WX Li XH Ma K Huo YB. Clinical treatment progress for large metacarpal and phalangeal bone defects. J Craniofac Surg. (2025) 36:e33–7. doi: 10.1097/SCS.0000000000010698, PMID: 39329505

[10] Gao H Huang J Wei Q He C. Advances in animal models for studying bone fracture healing. Bioengineering. (2023) 10:201. doi: 10.3390/bioengineering10020201, PMID: 36829695 PMC9952559

[11] Schindeler A Mills RJ Bobyn JD Little DG. Preclinical models for orthopedic research and bone tissue engineering. J Orthop Res. (2018) 36:832–40. doi: 10.1002/JOR.23824, PMID: 29205478

[12] Sparks DS Saifzadeh S Savi FM Dlaska CE Berner A Henkel J. A preclinical large-animal model for the assessment of critical-size load-bearing bone defect reconstruction. Nat Protoc. (2020) 15:877–924. doi: 10.1038/s41596-019-0271-2 32060491

[13] Beagan MLC Dreyer CH Jensen LK Jensen HE Andersen TE Overgaard S. The potential of sheep in preclinical models for bone infection research – a systematic review. J Orthop Translat. (2024) 45:120–31. doi: 10.1016/J.JOT.2024.02.002, PMID: 38524868 PMC10960093

[14] Bigorre N. Complications of osteosynthesis for long-finger metacarpal and phalanx fracture. Hand Surg Rehabil. (2024) 43:101746. doi: 10.1016/J.HANSUR.2024.101746, PMID: 38971225

[15] Adams JE Miller T Rizzo M. The biomechanics of fixation techniques for hand fractures. Hand Clin. (2013) 29:493–500. doi: 10.1016/j.hcl.2013.08.004, PMID: 24209948

[16] Hahn JA Witte TS Arens D Pearce A Pearce S. Double-plating of ovine critical sized defects of the tibia: a low morbidity model enabling continuous in vivo monitoring of bone healing. BMC Musculoskelet Disord. (2011) 12:214. doi: 10.1186/1471-2474-12-214, PMID: 21958221 PMC3195802

[17] Dozza B Salamanna F Baleani M Giavaresi G Parrilli A Zani L. Nonunion fracture healing: evaluation of effectiveness of demineralized bone matrix and mesenchymal stem cells in a novel sheep bone nonunion model. J Tissue Eng Regen Med. (2018) 12:1972–85. doi: 10.1002/term.2732, PMID: 30044550

[18] Viateau V Guillemin G Yang YC Bensaïd W Reviron T Oudina K. A technique for creating critical-size defects in the metatarsus of sheep for use in investigation of healing of long-bone defects. Am J Vet Res. (2004) 65:1653–7. doi: 10.2460/AJVR.2004.65.1653, PMID: 15631029

[19] Barcik J Ernst M Balligand M Dlaska CE Drenchev L Zeiter S. Short-term bone healing response to mechanical stimulation—a case series conducted on sheep. Biomedicine. (2021) 9:988. doi: 10.3390/biomedicines9080988, PMID: 34440192 PMC8392136

[20] Niemeyer P Fechner K Milz S Richter W Suedkamp NP Mehlhorn AT. Comparison of mesenchymal stem cells from bone marrow and adipose tissue for bone regeneration in a critical size defect of the sheep tibia and the influence of platelet-rich plasma. Biomaterials. (2010) 31:3572–9. doi: 10.1016/J.BIOMATERIALS.2010.01.085, PMID: 20153047

[21] Häger C Biernot S Buettner M Glage S Keubler LM Held N. The sheep grimace scale as an indicator of post-operative distress and pain in laboratory sheep. PLoS One. (2017) 12:e0175839. doi: 10.1371/journal.pone.0175839, PMID: 28422994 PMC5396914

[22] Hofmann-Fliri L Epari DR Schwyn R Zeiter S Windolf M. Biphasic plating – in vivo study of a novel fixation concept to enhance mechanobiological fracture healing. Injury. (2020) 51:1751–8. doi: 10.1016/j.injury.2020.04.032, PMID: 32536529

[23] Field J Ruthenbeck G. Qualitative and quantitative radiological measures of fracture healing. Vet Comp Orthop Traumatol. (2018) 31:001–9. doi: 10.3415/VCOT-17-03-0042, PMID: 29325186

[24] Richter H Plecko M Andermatt D Frigg R Kronen PW Klein K. Dynamization at the near cortex in locking plate osteosynthesis by means of dynamic locking screws. J Bone Joint Surg Am. (2015) 97:215. doi: 10.2106/JBJS.M.00529 25653321

[25] Decker S Krämer M Marten A-K Pfeifer R Wesling V Neunaber C. A nickel-titanium shape memory alloy plate for contactless inverse dynamization after internal fixation in a sheep tibia fracture model: a pilot study. Technol Health Care. (2015) 23:463–74. doi: 10.3233/THC-150912, PMID: 26409909

[26] Fugazzola MC Wever KE van de Lest C de Grauw J Salvatori D. Reporting of anaesthesia and pain management in preclinical large animal models of articular cartilage repair – a long way to go. Osteoarthr Cartil Open. (2022) 4:100261. doi: 10.1016/j.ocarto.2022.100261, PMID: 36475287 PMC9718186

[27] du Sert NP Ahluwalia A Alam S Avey MT Baker M Browne WJ. Reporting animal research: explanation and elaboration for the ARRIVE guidelines 2.0. PLoS Biol. (2020) 18:e3000411. doi: 10.1371/JOURNAL.PBIO.3000411 32663221 PMC7360025

[28] Mac Arthur Clark J Clifford P Jarrett W Pekow C. Communicating about animal research with the public. ILAR J. (2019) 60:34–42. doi: 10.1093/ILAR/ILZ007, PMID: 31095690

[29] Marcondes GDM Paretsis NF de Souza AF Ruivo MRBA Rego MAF Nóbrega FS. Locking compression plate fixation of critical-sized bone defects in sheep. Development of a model for veterinary bone tissue engineering. Acta Cir Bras. (2021) 36:601. doi: 10.1590/ACB360601 PMC8232063 34190837

[30] Wieding J Lindner T Bergschmidt P Bader R. Biomechanical stability of novel mechanically adapted open-porous titanium scaffolds in metatarsal bone defects of sheep. Biomaterials. (2015) 46:35–47. doi: 10.1016/j.biomaterials.2014.12.010, PMID: 25678114

[31] Colding-Rasmussen T Nikolaisen NK Horstman PF Petersen MM Hutchinson DJ Malkoch M. Customizable fracture fixation technique versus conventional metal locking plate: a comparative study of fracture fixation, stability and bone healing in an in vivo ovine phalangeal fracture model. Submitted Mater. (2025) 18:3359. doi: 10.3390/ma18143359, PMID: 40731567 PMC12298223

[32] EU. EU Directive—2010/63 of the European Parliament and of the Council of 22 September 2010 on the protection of animals used for scientific purposes. European Parliament and of the Council. (2010). Available online at: https://eur-lex.europa.eu/legal-content/EN/TXT/?uri=CELEX%3A32010L0063 (Accessed February 10, 2025)

[33] AWIN. (2015). AWIN welfare assessment protocol for sheep. doi: 10.13130/AWIN_SHEEP_2015

[34] Carroll GL Hartsfield SM. General anesthetic techniques in ruminants. Vet Clin N Am Food Anim Pract. (1996) 12:627–61. doi: 10.1016/S0749-0720(15)30391-1, PMID: 8916391

[35] Steffey EP. Some characteristics of ruminants and swine that complicate management of general anesthesia. Vet Clin North Am Food Anim Pract. (1986) 2:507–16. doi: 10.1016/S0749-0720(15)31203-2, PMID: 3539268

[36] Whelan DB Bhandari M Stephen D Kreder H Mckee MD Zdero R. Development of the radiographic union score for tibial fractures for the assessment of tibial fracture healing after intramedullary fixation. J Trauma Inj Infect Crit Care. (2010) 68:629–32. doi: 10.1097/TA.0B013E3181A7C16D, PMID: 19996801

[37] Lane JM Sandhu HS. Current approaches to experimental bone grafting. Orthop Clin North Am. (1987) 18:213–25. doi: 10.1016/S0030-5898(20)30385-0 3550572

[38] McLennan KM Rebelo CJB Corke MJ Holmes MA Leach MC Constantino-Casas F. Development of a facial expression scale using footrot and mastitis as models of pain in sheep. Appl Anim Behav Sci. (2016) 176:19–26. doi: 10.1016/j.applanim.2016.01.007

[39] Lindegaard C Thomsen MH Larsen S Andersen PH. Analgesic efficacy of intra-articular morphine in experimentally induced radiocarpal synovitis in horses. Vet Anaesth Analg. (2010) 37:171–85. doi: 10.1111/j.1467-2995.2009.00521.x, PMID: 20230568

[40] Stillman MW Whittaker AL. Use and efficacy of analgesic agents in sheep (*Ovis aries*) used in biomedical research. J Am Assoc Lab Anim Sci. (2019) 58:755–66. doi: 10.30802/AALAS-JAALAS-19-000036, PMID: 31604483 PMC6926396

[41] López-Arjona M Tecles F Mateo SV Contreras-Aguilar MD Martínez-Miró S Cerón JJ. Measurement of cortisol, cortisone and 11β-hydroxysteroid dehydrogenase type 2 activity in hair of sows during different phases of the reproductive cycle. Vet J. (2020) 259-260:105458. doi: 10.1016/j.tvjl.2020.105458, PMID: 32553232

[42] Franco-Martínez L Muñoz-Prieto A Contreras-Aguilar MD Želvytė R Monkevičienė I Horvatić A. Changes in saliva proteins in cows with mastitis: a proteomic approach. Res Vet Sci. (2021) 140:91–9. doi: 10.1016/j.rvsc.2021.08.008, PMID: 34418789

[43] Schmidt EM d S Fachiolli DF de Oliveira RM Almeida FA Pariz CM de Lima Meirelles PR. Changes in serum thiol-disulphide homeostasis in sheep with gastrointestinal nematodes. Animals. (2021) 11:2856. doi: 10.3390/ani11102856 34679878 PMC8532846

[44] Nagy DW Pugh DG. Handling and examining sheep and goats. In Pugh DG Baird AN, (Eds) Sheep Goat Med. (Second edition) (2012):1–17. doi: 10.1016/B978-1-4377-2353-3.10001-0, PMID: 40875055

[45] Ronca A Guarino V Raucci MG Salamanna F Martini L Zeppetelli S. Large defect-tailored composite scaffolds for in vivo bone regeneration. J Biomater Appl. (2014) 29:715–27. doi: 10.1177/0885328214539823, PMID: 24951457

[46] Pape D Madry H. The preclinical sheep model of high tibial osteotomy relating basic science to the clinics: standards, techniques and pitfalls. Knee Surg Sports Traumatol Arthrosc. (2013) 21:228–36. doi: 10.1007/s00167-012-2135-y 22820740

[47] Williams NJ Streeter RN. Nonpathological phalangeal fractures in cattle: 17 cases (2004–2020). J Am Vet Med Assoc. (2022) 260:350–6. doi: 10.2460/JAVMA.21.05.0265, PMID: 34890363

[48] Ceciliani F Ceron JJ Eckersall PD Sauerwein H. Acute phase proteins in ruminants. J Proteome. (2012) 75:4207–31. doi: 10.1016/J.JPROT.2012.04.004, PMID: 22521269

[49] Jacobsen S Nielsen JV Kjelgaard-Hansen M Toelboell T Fjeldborg J Halling-Thomsen M. Acute phase response to surgery of varying intensity in horses: a preliminary study. Vet Surg. (2009) 38:762–9. doi: 10.1111/J.1532-950X.2009.00564.X, PMID: 19674420

[50] Fell LR Shutt DA. Behavioural and hormonal responses to acute surgical stress in sheep. Appl Anim Behav Sci. (1989) 22:283–94. doi: 10.1016/0168-1591(89)90023-3

[51] Contreras-Aguilar MD Escribano D Quiles A López-Arjona M Cerón JJ Martínez-Subiela S. Evaluation of new biomarkers of stress in saliva of sheep. Animal. (2019) 13:1278–86. doi: 10.1017/S1751731118002707, PMID: 30362447

[52] Barry S. Non-steroidal anti-inflammatory drugs inhibit bone healing: a review. Vet Comp Orthop Traumatol. (2010) 23:385–92. doi: 10.3415/VCOT-10-01-0017, PMID: 20830450

[53] Schröter L Kaiser F Preißler AL Wohlfahrt P Küppers O Gbureck U. Ready-to-use and rapidly biodegradable magnesium phosphate bone cement: in vivo evaluation in sheep. Adv Healthc Mater. (2023) 12:e2300914. doi: 10.1002/adhm.202300914, PMID: 37224104 PMC11468836

[54] Dreyer CH Jørgensen NR Overgaard S Qin L Ding M. Vascular endothelial growth factor and mesenchymal stem cells revealed similar bone formation to allograft in a sheep model. Biomed Res Int. (2021) 2021:6609. doi: 10.1155/2021/6676609, PMID: 33763484 PMC7946458

[55] Axelsen MG Overgaard S Jespersen SM Ding M. Comparison of synthetic bone graft ABM/P-15 and allograft on uninstrumented posterior lumbar spine fusion in sheep. J Orthop Surg Res. (2019) 14:2. doi: 10.1186/s13018-018-1042-4, PMID: 30606209 PMC6318885

[56] Molony V Kent JE. Assessment of acute pain in farm animals using behavioral and physiological measurements. (1997). 75:266–272. 10.2527/1997.751266x 9027575

[57] Willie BM Bloebaum RD Bireley WR Bachus KN Hofmann AA. Determining relevance of a weight-bearing ovine model for bone ingrowth assessment. J Biomed Mater Res A. (2004) 69:567–76. doi: 10.1002/jbm.a.30038, PMID: 15127404

[58] Cunningham BW Brooks DM Rolle NP Weiner DA Wang W. An investigational time course study of titanium plasma spray on osseointegration of PEEK and titanium implants: an in vivo ovine model. Spine J. (2024) 24:721–9. doi: 10.1016/j.spinee.2023.10.005, PMID: 37875243

[59] Lu Y Lee JS Nemke B Graf BK Royalty K Illgen R. Coating with a modular bone morphogenetic peptide promotes healing of a bone-implant gap in an ovine model. PLoS One. (2012) 7:e50378. doi: 10.1371/journal.pone.0050378, PMID: 23185610 PMC3503930

[60] Steagall PV Bustamante H Johnson CB Turner PV. Pain management in farm animals: focus on cattle, sheep and pigs. Animals. (2021) 11:1483. doi: 10.3390/ANI11061483 34063847 PMC8223984

[61] Darwin C. The expression of the emotions in man and animals. London: John Murray (1872).

[62] Stafford KJ. Recognition and assessment of pain in ruminants In: Egger CM Love L Doherty T (eds) Pain management in veterinary practice. John Wiley & Sons, Ltd: Chichester, UK (2013). 349–57.

[63] Feighelstein M Luna SP Silva NO Trindade PE Shimshoni I van der Linden D. Comparison between AI and human expert performance in acute pain assessment in sheep. Sci Rep. (2025) 15:626. doi: 10.1038/s41598-024-83950-y, PMID: 39754012 PMC11698723

