# Supplementary material for: First steps in the development of an ovine proximal phalanx fracture and bone defect model: a study of animal welfare and bone healing
Source: Front Vet Sci. 2025 Oct 8;12:1662553. doi: 10.3389/fvets.2025.1662553 (PMC12540181; doi:10.3389/fvets.2025.1662553)
Supplement: Supplementary file 1 [file Data_Sheet_1.PDF]

Supplementary Material

1 Supplementary Figures and Tables

1.1 Supplementary Figures

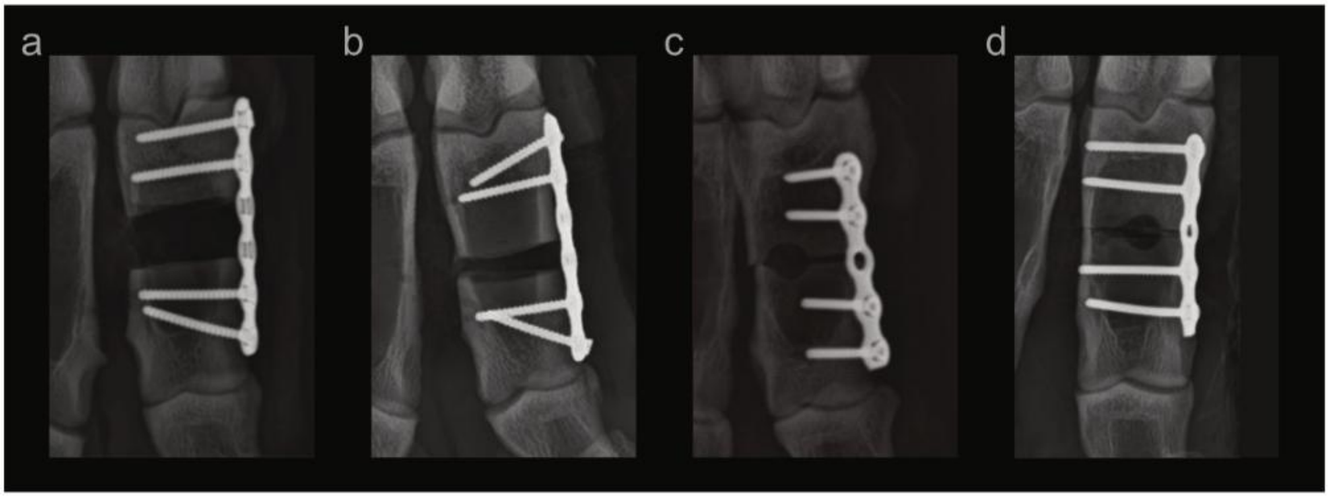

Supplementary Figure 1: Representative intraoperative radiographs from; a) ostectomy 6 mm gap, b) ostectomy 3 mm gap, c) osteotomy subgroup A with unicortical screws, and d) osteotomy subgroup B with bicortical screws.

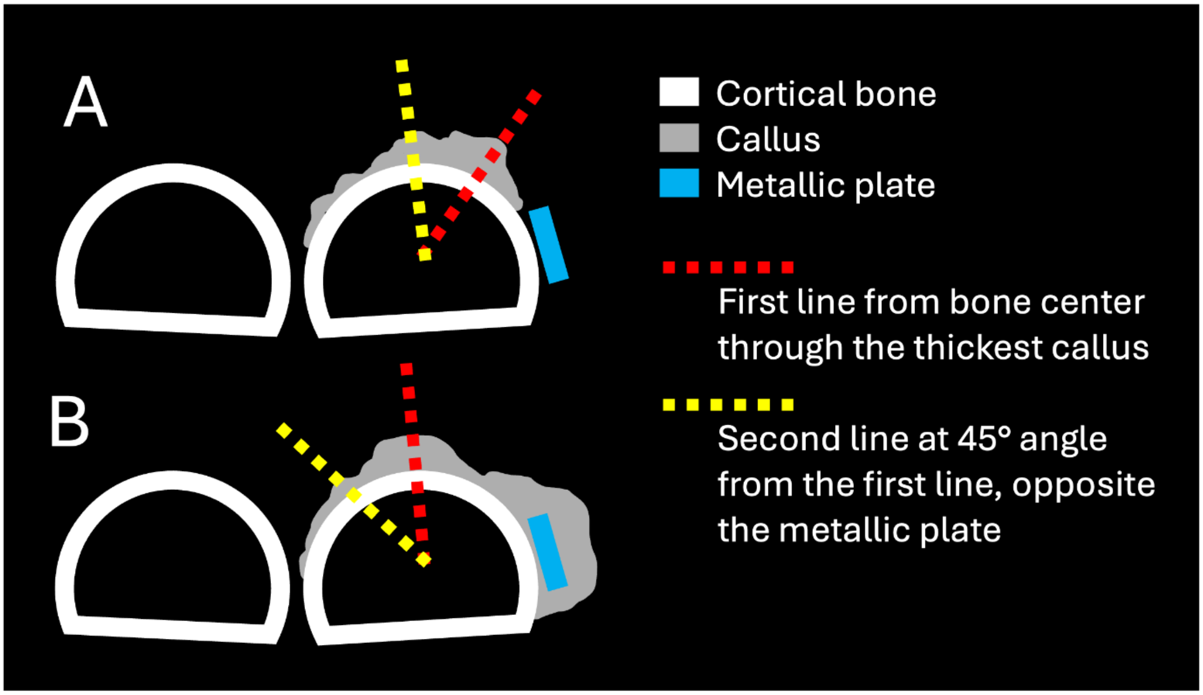

Supplementary Figure 2: Schematic transverse CT images of the proximal (A) and distal (B) fracture ends of the proximal phalanges of digits 3 (left) and 4 (right). Cortical bone (white), callus (gray), and metallic plate (blue) are shown. Callus thickness was measured from the outer cortex and through the thickest point of the callus, along a line extending from the center of the bone (red dotted line). A second measurement was taken along a line at 45 ° to the first line and positioned opposite the plate (yellow dotted line). Callus surrounding the plate, as shown in B, was excluded to avoid bias; the second-thickest region was used instead. Both measurements were recorded at the proximal and distal bone fragment, and the mean was calculated and reported.

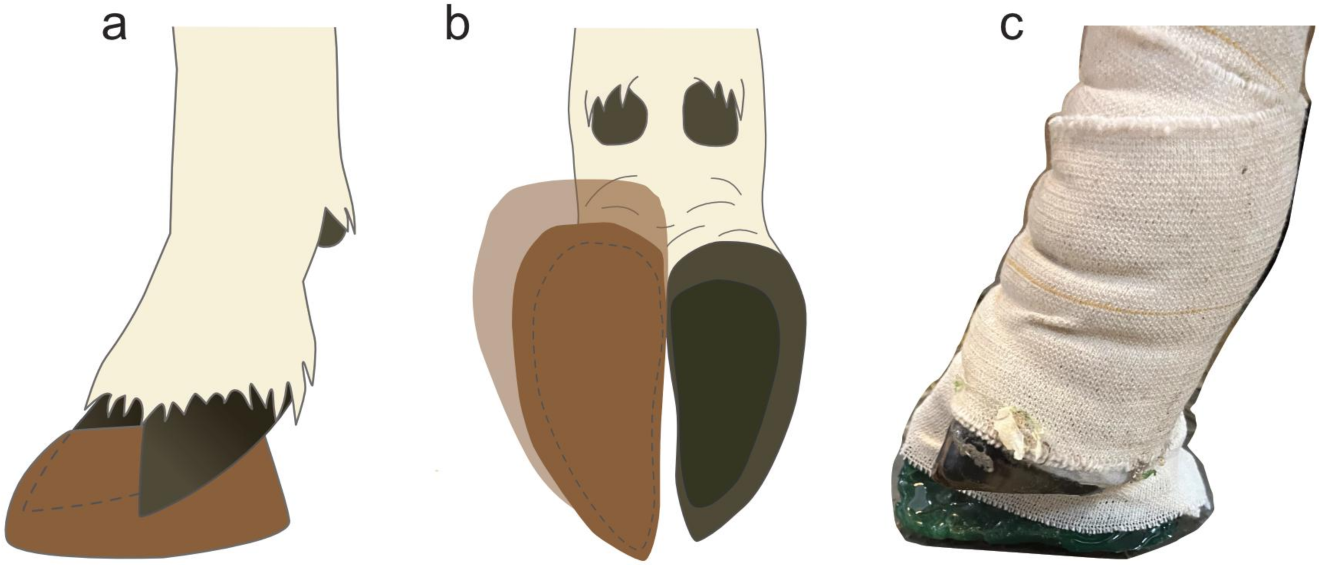

Supplementary Figure 3: Schematic drawing of the distal limb of a sheep with hoof block (brown) applied on the medial digit, a) lateral view, with the lateral digit offloaded and b) palmar view, the brown area showing the hoof block and the transparent brown area indicating a modification of the hoof block with an increased weight bearing surface. c) Image of a distal limb with a hoof block.

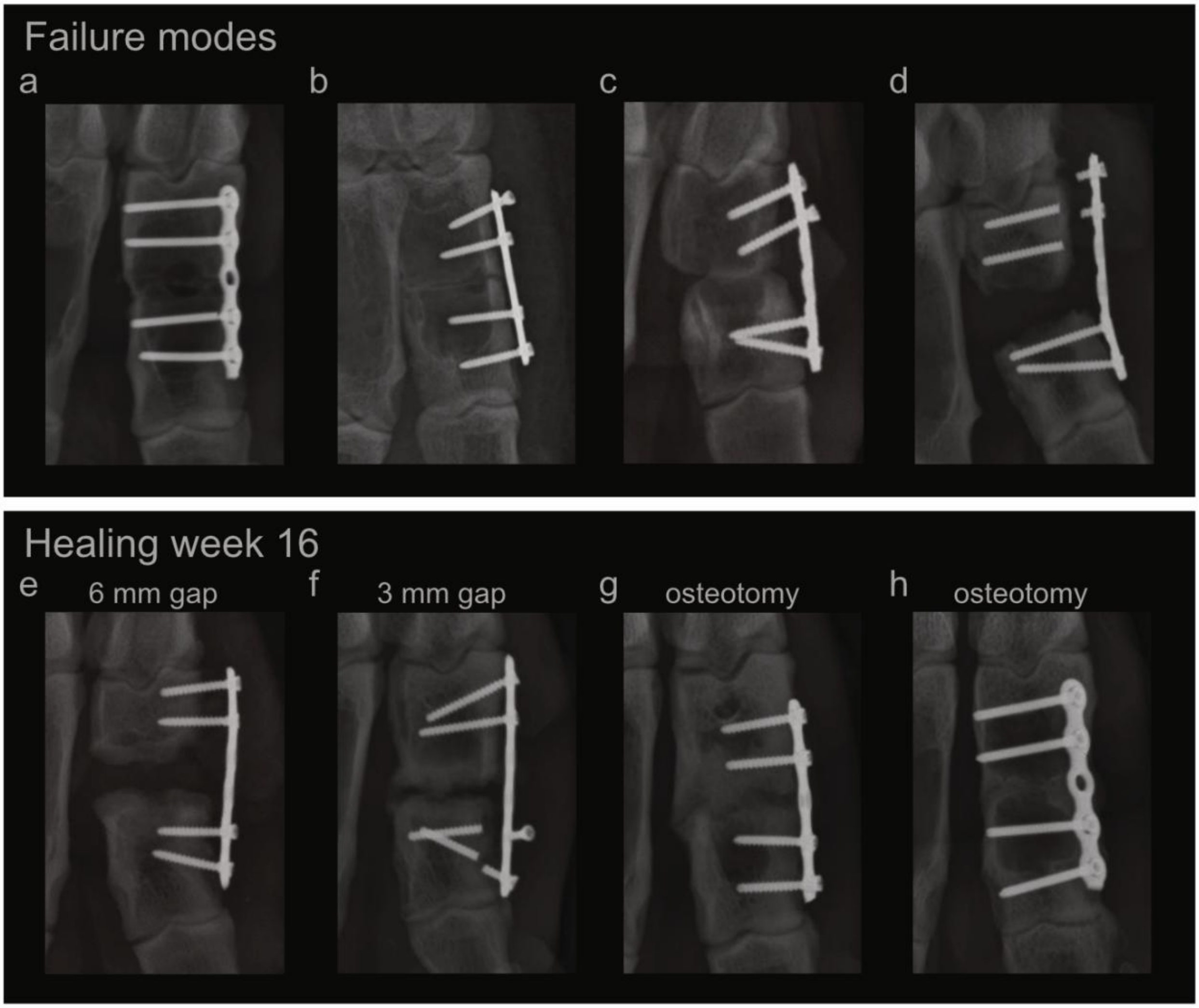

Supplementary Figure 4: Illustration of failure modes of the implants (a-d); a) screw breakage (osteotomy, subgroup B), b) screw loosening (osteotomy, subgroup A), c) screw loosening and plate dislodgment (ostectomy, 6 mm gap), and d) screw breakage and plate dislodgment (ostectomy, 6 mm gap). Representative healing status' at week 16 (e-h); e) ostectomy 6 mm gap demonstrating no healing in the fracture gap and rounding of the fracture ends, f) ostectomy 3 mm gap demonstrating initial callus formation across the fracture gap, g) osteotomy subgroup A demonstrating bone healing with callus formation (unicortical screw fixation) and e) osteotomy subgroup B demonstrating bone healing with callus formation (bicortical screw fixation).

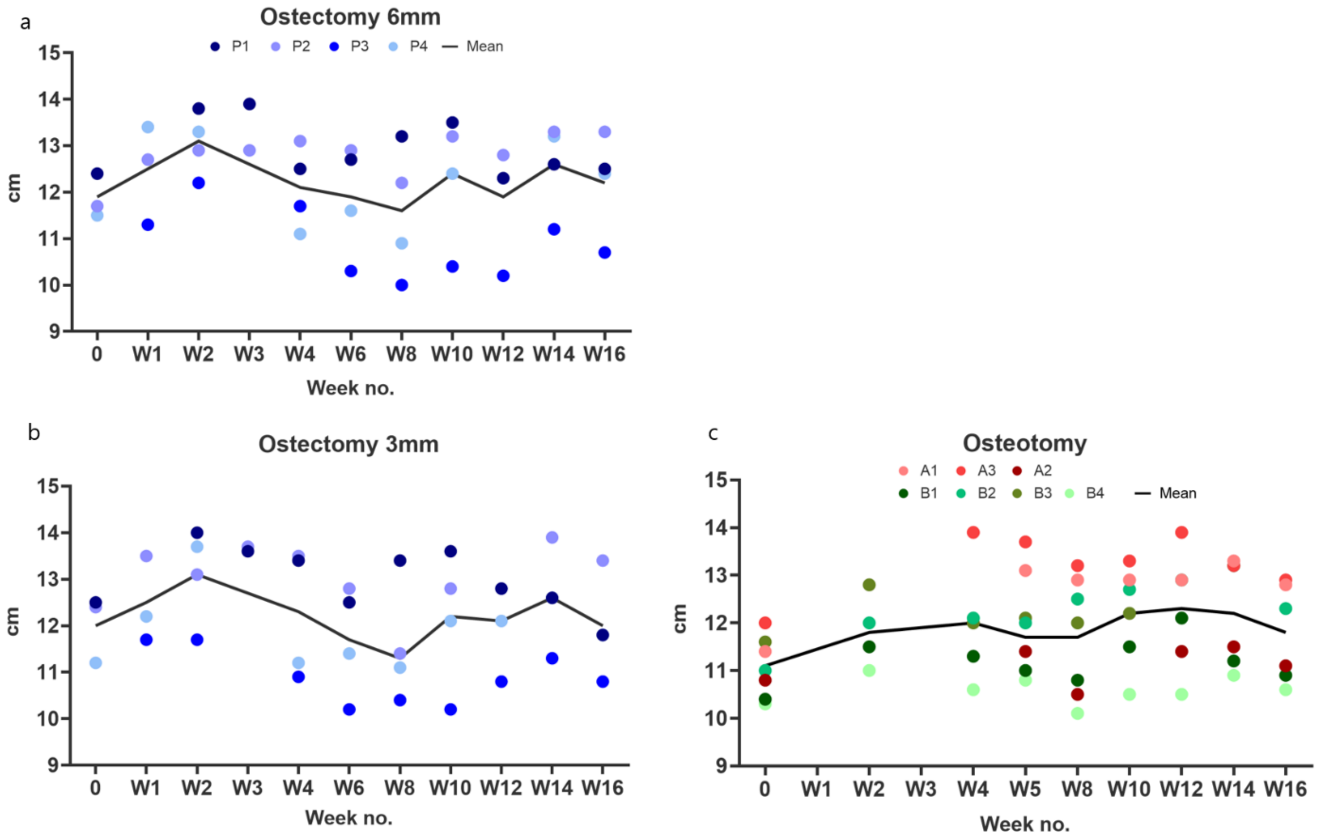

The number of sheep measured varied; for the ostectomy group: day 0 (n = 3), day 7 (n = 3), week 3 (n = 2, consequently, not included in the mean), and week 4-16 (n = 4). For the osteotomy group: day 0 (n = 7), day 11 (n = 4), week 4 (n = 5), week 5 (n = 6), week 8 (n = 7), week 10 -16 (n = 6). At week 8 when elastic bandages were removed. The hair of the legs was cut on the first and the last measurements.

Supplementary Figure 5: Circumference of the four sheep in the ostectomy study with a 6 mm gap (a) and 3 mm gap (b) and the seven sheep in the osteotomy study (c).
